# Using high-throughput sequencing to investigate the factors structuring genomic variation of a Mediterranean grasshopper of great conservation concern

**DOI:** 10.1038/s41598-018-31775-x

**Published:** 2018-09-07

**Authors:** María José González-Serna, Pedro J. Cordero, Joaquín Ortego

**Affiliations:** 1 Grupo de Investigación de la Biodiversidad Genética y Cultural, Instituto de Investigación en Recursos Cinegéticos – IREC – (CSIC, UCLM, JCCM), Ronda de Toledo, 12, E-13071 Ciudad Real, Spain; 2 Department of Integrative Ecology, Estación Biológica de Doñana – EBD – (CSIC), Avda. Américo Vespucio, 26, E-41092 Seville, Spain

## Abstract

Inferring the demographic history of species is fundamental for understanding their responses to past climate/landscape alterations and improving our predictions about the future impacts of the different components of ongoing global change. Estimating the time-frame at which population fragmentation took place is also critical to determine whether such process was shaped by ancient events (e.g. past climate/geological changes) or if, conversely, it was driven by recent human activities (e.g. habitat loss). We employed genomic data (ddRAD-Seq) to determine the factors shaping contemporary patterns of genetic variation in the Iberian cross-backed grasshopper *Dociostaurus crassiusculus*, an endangered species with limited dispersal capacity and narrow habitat requirements. Our analyses indicate the presence of two ancient lineages and three genetic clusters resulted from historical processes of population fragmentation (~18–126 ka) that predate the Anthropocene. Landscape genetic analyses indicate that the limits of major river basins are the main geographical feature explaining large-scale patterns of genomic differentiation, with no apparent effect of human-driven habitat fragmentation. Overall, our study highlights the importance of detailed phylogeographic, demographic and spatially-explicit landscape analyses to identify evolutionary significant units and determine the relative impact of historical *vs*. anthropogenic factors on processes of genetic fragmentation in taxa of great conservation concern.

## Introduction

Inferring the evolutionary and demographic history of species and populations is fundamental for understanding how they were impacted by past environmental and landscape alterations and anticipating their responses to different components of global change such as climatic variations^1–3^, habitat loss^4^ or the emergence of infectious diseases^5^. Many organisms show nowadays highly fragmented distributions due to a natural patchy distribution of their particular habitats^6,7^ or as consequence of their originally continuous populations became isolated due to habitat fragmentation driven by human activities^8,9^ or past climatic/geological events^10,11^. The genetic, ecological and evolutionary consequences of severe population fragmentation are numerous, including alteration of selective pressures, genetic erosion, inbreeding, accumulation of deleterious mutations, reduced evolutionary potential and, ultimately, increased risk of extinction^12,13^. For these reasons, the study of population fragmentation is a major area of research for conservation biologists and geneticists, and particular attention has been paid to taxa forming small populations and presenting narrow distributions, low dispersal capabilities, and specific habitat requirements^14,15^.

Estimating the time-frame at which population fragmentation took place is critical to determine whether such process was driven by historical processes that predate the Anthropocene or if, conversely, it is a direct consequence of human activities^16,17^. This has important implications to inform on ground conservation practices^18^. If recent human-induced habitat fragmentation is identified as the main driver of population genetic structure and disruption of gene flow, then conservation practices should focus on restoring population connectivity either creating corridors to dispersal or assisting gene flow to avoid the long-term negative consequences of inbreeding and loss of genetic diversity^19,20^. If, instead, population genetic structure was driven by ancient processes, then the different clades, lineages or genetic clusters might represent evolutionary significant units (ESUs) with particular local adaptations that deserve to be managed independently to maximize the protection of both vicariant and adaptive diversity^18,21,22^. Beyond the temporal scale of population divergence, identifying the proximate factors shaping contemporary patterns of genetic structure is also fundamental to understand how organisms interact with the different components of the landscape^23^. Genetic and spatial information has been successfully integrated to infer dispersal routes across different habitat types^20^, identify natural barriers to dispersal (e.g. rivers^24^, topography^25^, geology^26^) and determine the consequences of human activities on disrupting gene flow of natural populations (e.g. agriculture^27,28^, infrastructures^29^). For this reason, testing alternative spatially-explicit scenarios of population connectivity under a landscape genetic framework can help to determine the relative role of human and natural barriers to gene flow on structuring present-day patterns of genetic variation^30^. This takes particular relevance in the case of specialist taxa with patchy distributions, as identifying contemporary barriers to gene flow and cryptic corridors to dispersal is crucial to establish management practices aimed to restore or enhance connectivity among remnant populations^20^.

The Iberian Peninsula constitutes an important biodiversity hotspot, with high species richness, rates of endemism and levels of intra-specific genetic diversity^31–33^. Explanations for the high diversity of the Iberian Peninsula include its historically high climatic stability^34^, the low impact of Pleistocene glaciations in comparison with northern temperate areas^35^, its current proximity and Miocene connection with North Africa and other Mediterranean regions^10,36^, and the presence of deep environmental gradients and a complex topography^34,37^. Despite its high biodiversity and conservation value, the Mediterranean region has been exposed to millennia of strong human intervention^27,34^ that have reduced the original extent of its primary vegetation by ~95%^33^. This region is also predicted to be impacted by intense effects of climate change and experience distributional shifts and remarkable range contractions in many taxa^38,39^. Both severe habitat loss and climate warming represent serious threats for many taxa with small and highly fragmented distributions that deal with important difficulties for maintaining viable populations and face risk of extinction^40,41^. Thus, understanding the evolutionary history, demographic trends, and interactions with landscape heterogeneity of these taxa is critical for establishing effective conservation policies and informed management practices that ensure their long-term persistence^42,43^.

In this study, we use genomic data to infer the processes structuring genetic variation in the Iberian cross-backed grasshopper *Dociostaurus crassiusculus* (Pantel, 1886), a species of great conservation concern that has been recently catalogued as “endangered” in the Red List of European Orthoptera^44^. The taxonomic position of this species was controversial and according to morphological criteria it has been considered for a long time a subspecies of the Asian *Dociostaurus kraussi* (Ingenitskii, 1897)^45–47^. A recent re-evaluation of the taxonomic status of the genus using genetic data has supported the presence of two well recognized species in concordance with their disjunct distributions: *D*. *crassiusculus* in the Iberian Peninsula and *D*. *kraussi* in Asia^48^. The full species status of *D*. *crassiusculus* makes it of higher conservation concern provided that the very few known populations of the species persist in highly isolated pockets of habitat embedded in an expansive matrix of unsuitable areas^3^. The species is currently distributed in central-southeast Iberia, mostly occupying pseudo-steppe habitats with halophytic plant communities linked to gypsum or hypersaline soils^3^. These narrow habitat requirements, together with the reduced flying capacity of the species and the progressive loss of its natural habitat by human activities, has led that all populations of *D*. *crassiusculus* are nowadays extremely fragmented and at high risk of extinction by stochastic phenomena^3,49^.

Here, we employ restriction-site-associated DNA sequencing (ddRAD-seq), coalescent-based simulations and a landscape genetics framework to evaluate alternative demographic scenarios, estimate the timing of population fragmentation, and infer the processes shaping contemporary patterns of genetic structure across all known populations of *D*. *crassiusculus*. Specifically, we first used genomic data to analyse the spatial genetic structure of extant populations of the species, identify main lineages and establish their phylogenomic relationships, and define hierarchical units for conservation and management^18,21^. Second, we tested alternative coalescent-based demographic and migration models to infer spatial patterns and rates of inter-population gene flow, estimate the timing of population fragmentation at different spatial scales and, ultimately, determine whether the genetic structure of the species is a consequence of ancient events (e.g. past climate or geological changes) or if, conversely, it is compatible with human-driven population fragmentation^16^. Finally, we generated alternative isolation-by-resistance (IBR) scenarios of population connectivity within a spatially-explicit framework to identify the specific landscape features shaping genetic differentiation in the species and unravel the relative importance of natural (topography, lithology, limits of main river basins) *vs*. anthropogenic (habitat loss) processes of genetic fragmentation.

## Results

### Genomic data and genetic statistics

A total of 91,666,732 reads were obtained for the 35 genotyped individuals of *D*. *crassiusculus*. The number of reads per individual (mean ± SD = 2,619,049 ± 841,054 reads) before and after different quality filtering steps is shown in Supplementary Fig. S1. The datasets obtained with Stacks for *p* = 2 and *p* = 4 contained 80,534 and 65,459 unlinked SNPs, respectively. The datasets obtained with PyRAD for *W*_*clust*_ = 90% and *MinCov* = 11 and 23 contained 32,424 and 18,442 unlinked SNPs, respectively; and for *W*_*clust*_ = 95% and *MinCov* = 11 and 23 contained 42,053 and 23,628 unlinked SNPs, respectively. Population genetic statistics (*P*, *π*, *H*_*O*_, *H*_*E*_ and *F*_IS_) calculated with Stacks for all positions (polymorphic and non-polymorphic) and considering loci that were represented in at least two (*p* = 2) and four populations (*p* = 4) and the 50% of individuals within populations (*r* = 0.5) are presented in Supplementary Table S1. Pair-wise *F*_ST_ values ranged from 0.063 to 0.237 and all were significantly different from zero based on 100 permutations (*P* < 0.05; Supplementary Table S2).

### Population genetic structure

**Figure 1:**
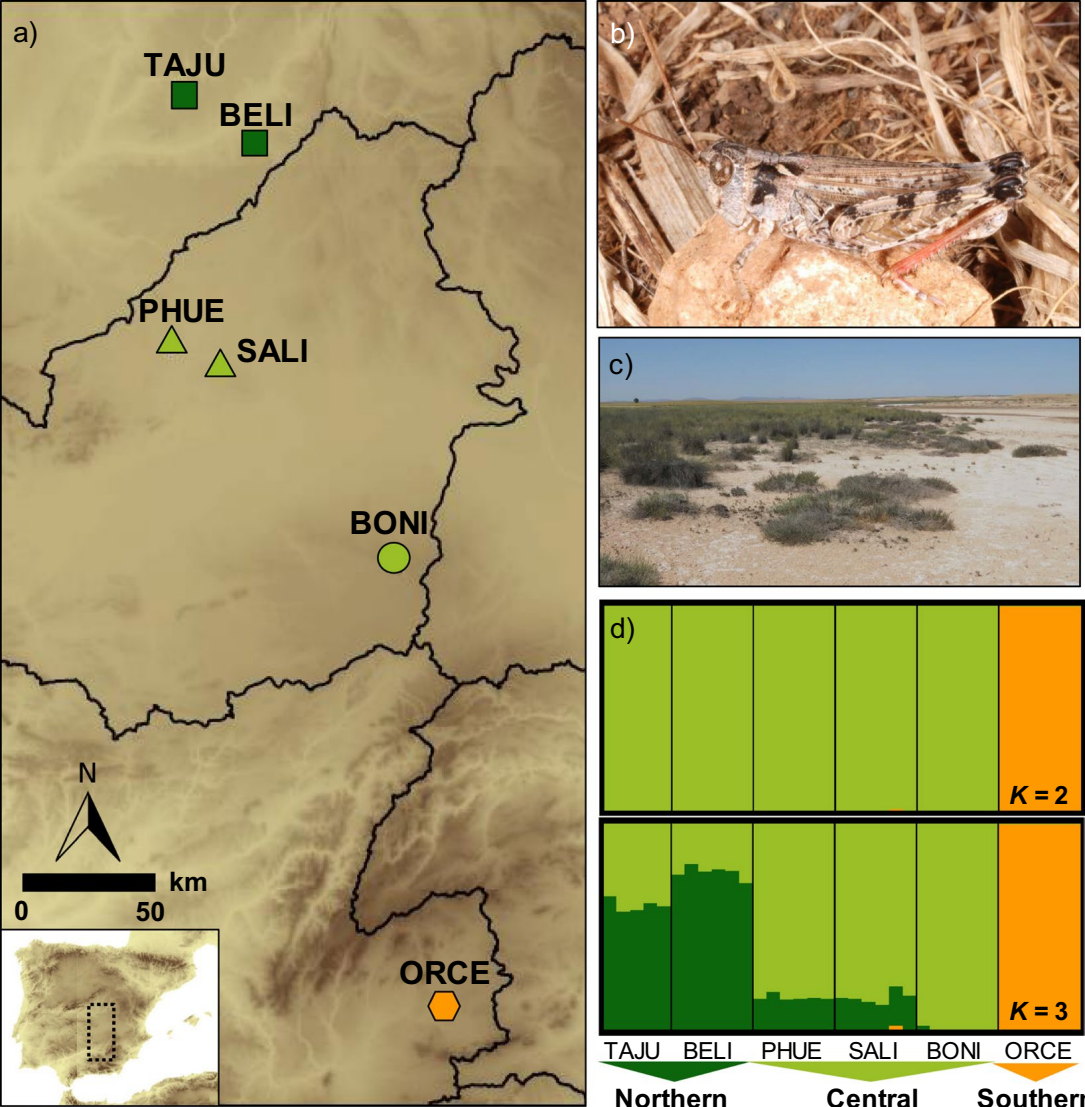
(**a**) Geographical location and genetic structure of the studied populations of the Iberian cross-backed grasshopper *Dociostaurus crassiusculus*. Brown shading in the map represents elevation, with darker areas corresponding to higher altitudes. Black lines show the boundaries of the main river basins separating the three population groups: Northern (dark green), Central (light green) and Southern (orange). (**b**) Male of *D*. *crassiusculus*. (**c**) Typical habitat of the species, with gypsophilous grounds and wastelands with halophytic vegetation. (**d**) Barplots showing the genetic assignments of the different individuals based on the Bayesian method implemented in the program Structure for *K* = 2 and *K* = 3. Each individual is represented by a vertical bar, which is partitioned into *k* coloured segments showing the individual’s probability of belonging to the cluster with that colour. Thin vertical black lines separate individuals from different populations. These analyses are based on a random subset of 10,000 unlinked SNPs obtained with Stacks (*p* = 4).

**Figure 2:**
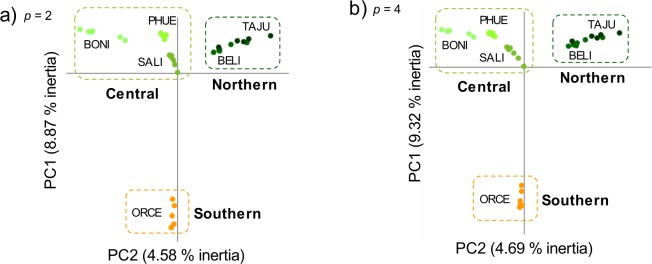
Principal component analyses (PCA) of genetic variation for populations of *D*. *crassiusculus*. Analyses are based on SNP datasets obtained with Stacks considering different filtering parameters: (**a**) 80,534 unlinked SNPs for *p* = 2; and (**b**) 65,459 unlinked SNPs for *p* = 4. Dotted-line rectangles group main population clusters. Population codes are described in Table 4.

Structure analyses based on a random subset of unlinked 10,000 SNPs from six different datasets obtained with Stacks and PyRAD considering different filtering/clustering parameters (see Supplementary Methods for further details), always identified *K* = 2 as the most likely clustering solution according with the Δ*K* criterion (Supplementary Figs 2 and 3). The two clusters presented no signature of genetic admixture and split the southernmost population (ORCE) from the remainder of the populations (Fig. 1d). Structure analyses for *K* = 3 divided Northern (TAJU-BELI) and Central populations (PHUE-SALI-BONI) in two different genetic clusters, but in this case the geographically closer populations (TAJU-BELI and PHUE-SALI) showed a considerable degree of genetic admixture (~25%) (Fig. 1d and Supplementary Fig. S2). The results obtained with Structure were in agreement with those obtained from Principal Component Analyses (PCA), in which PC1 split the Southern population (ORCE) from the remainder of the populations, and PC2 separated Northern (TAJU-BELI) from Central populations (PHUE-SALI-BONI) (Fig. 2 and Supplementary Fig. S4).

### Phylogenomic inference

**Figure 3:**
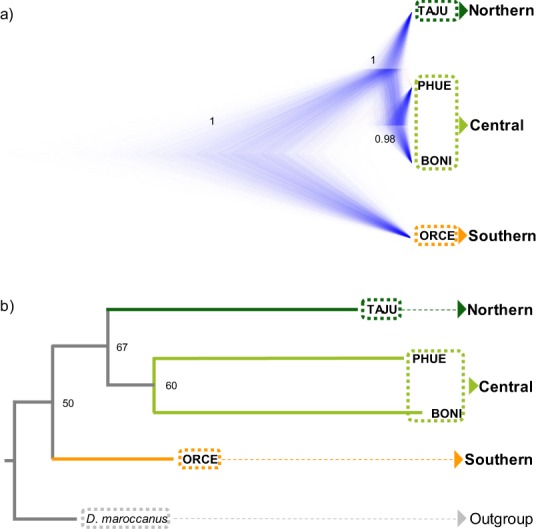
Phylogenetic trees inferred with (**a**) Snapp and (**b**) SVDQuartets considering four populations representative of the main geographical areas (populations separated >80 km) and the three genetic groups identified by Principal Component Analyses (PCA) and Bayesian clustering analyses in Structure (Northern, Central and Southern populations). Bayesian posterior probabilities (for Snapp) and bootstrapping support values (for SVDQuartets) are indicated on the nodes. Population codes are described in Table 4.

Phylogenomic relationships among populations inferred by Snapp were well-resolved and nodes presented high posterior probabilities (Fig. 3a). In agreement with analyses of genetic structure (Structure and PCAs), Snapp analyses supported an earlier split of ORCE from the remainder of the populations, which in turn divided into Northern (TAJU) and Central populations (PHUE and BONI) (Fig. 3a). Analyses considering different current and ancestral population sizes (*α* = 2; *β* = 200 or *α* = 2; *β* = 20,000) and different population combinations for Central and Northern populations (i.e. BELI-SALI, BELI-PHUE, and TAJU-SALI) yielded analogous results (data not shown)^50^. The best tree from SVDquartets yielded the same topology than Snapp, but the relationships among populations were not well resolved (bootstrap support values <70%) probably as a result of inter-population gene flow or incomplete lineage sorting (Fig. 3b).

### Coalescent-based demographic models

**Figure 4:**
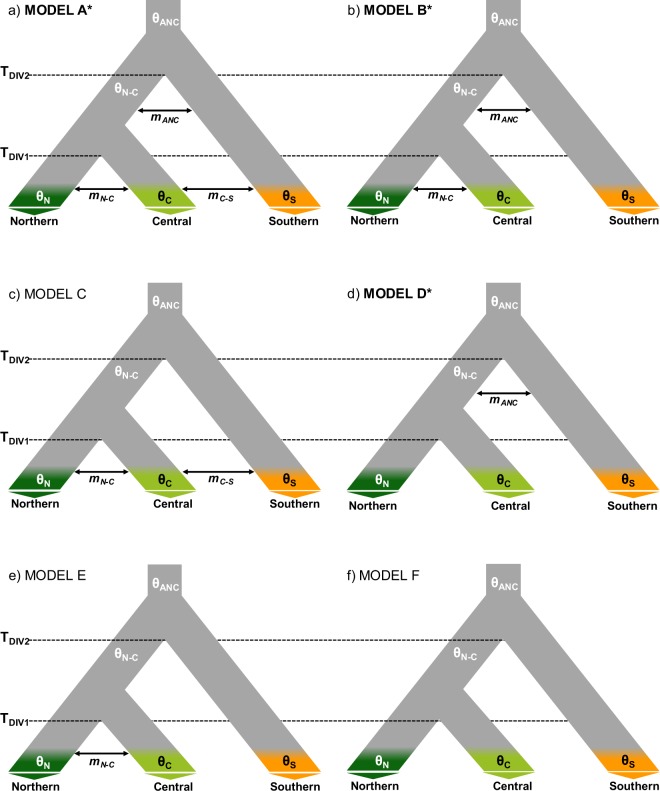
Alternative migration models tested using Fastsimcoal2. Parameters include ancestral (θ_ANC_, θ_N-C_) and contemporary (θ_N_, θ_C_, θ_S_) effective population sizes, timing of population split (T_DIV2_, T_DIV1_), and migration rates (*m*) between different pairs of populations. An asterisk and bold type indicate the three best supported migration models (see the Results section and Table 1 for more details).

Fastsimcoal2 analyses supported models A, B and D (Fig. 4) as the best-fitting and statistically equivalent models (ΔAIC < 2.00; Table 1). These three migration models have in common that all of them consider gene flow between ancestral populations (*m*_ANC_) (Fig. 4). Although analogous models without ancestral migration were tested (models C, E and F; Fig. 4), they were poorly supported (Table 1). Demographic parameters estimated under the three best supported models (A, B, and D) and their weighted averages are presented in Table 2. Considering a 1-year generation time for *D*. *crassiusculus*^3^, Fastsimcoal2 analyses showed that the division between the Southern and Northern-Central populations (T_DIV2_) occurred ~126 ka (95% CIs: 90–197 ka), probably during the Eemian Interglacial period (115–130 ka) (Table 2). The weighted average estimate yielded by Fastsimcoal2 for the more recent split between Northern and Central populations (T_DIV1_) indicate that this event took place ~17 ka (95% CIs: 11–24 ka), around the last glacial maximum (LGM; 20 ka) (Table 2). Gene-flow estimates were low and the migration rate (*m*) inferred between Central and Southern populations (*m*_C-S_) was nearly an order of magnitude lower than the migration rate between Northern and Central populations (*m*_N-C_) and between ancestral populations (*m*_ANC_) after the first population split (T_DIV2_) (Table 2).

**Table 1:** Comparison of alternative migration models (detailed in Fig. 4) tested using Fastsimcoal2.

Model	ln*L*	*k*	AIC	ΔAIC	*ω*_*i*_
**A**	**−19**,**539**.**06**	**11**	**39**,**100**.**12**	**1**.**76**	**0**.**19**
**B**	**−19**,**539**.**18**	**10**	**39**,**098**.**36**	**0**.**00**	**0**.**46**
C	−19,546.21	10	39,112.43	14.07	0.00
**D**	**−19**,**540**.**48**	**9**	**39**,**098**.**97**	**0**.**61**	**0**.**34**
E	−19,566.71	9	39,151.42	53.06	0.00
F	−19,567.69	8	39,151.38	53.02	0.00

For each model, the table shows the maximum likelihood estimate (ln*L*), the number of parameters (*k*), the Akaike’s information criterion score (AIC), the difference in AIC value of each model from that of the strongest model (ΔAIC), and AIC weight (ω_i_). Best-supported equivalent models (ΔAIC < 2) are indicated in bold (Fig. 4).

**Table 2:** Parameters inferred from coalescent simulations with Fastsimcoal2 under the three best supported migration models (see Fig. 4 for details).

Parameter	Model A	Model B	Model D	Model average (95% CIs)
θ_ANC_	99,783	95,231	111,992	101,840 (48,527–121,923)
θ_N-C_	148,756	150,712	195,197	165,446 (134,574–201,611)
θ_N_	163,990	166,040	130,914	153,687 (136,724–200,599)
θ_C_	102,756	118,736	78,941	102,010 (91,758–131,587)
T_DIV1_	31,995	19,268	4,507	16,795 (11,509–23,731)
T_DIV2_	152,866	156,242	68,431	125,711 (90,218–197,204)
*m*_ANC_	2.25 × 10^–05^	1.45 × 10^−05^	5.30 × 10^−06^	1.30 × 10^−05^ (8.37 × 10^−06^–1.50 × 10^−04^)
*m*_N-C_	3.86 × 10^−05^	3.30 × 10^−05^	—	3.47 × 10^−05^ (2.69 × 10^–05^–3.99 × 10^−05^)
*m*_C-S_	1.83 × 10^−06^	—	—	1.83 × 10^−06^ (3.57 × 10^−07^–2.97 × 10^−06^)

The effective population size of one population (ORCE: θ_S_) was calculated from nucleotide diversity estimates and fixed in the different models to enable the estimation of other parameters (see the Methods section). Table shows point estimates under each model and model averaged estimates with lower and upper 95% confidence intervals in parenthesis. Estimates of time are given in units of generations.

### Landscape genetic analyses

**Figure 5:**
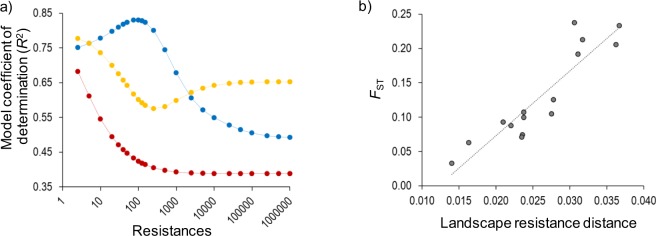
(**a**) Coefficient of determination (*R*^2^) for models analysing genetic differentiation (*F*_ST_) in relation with resistance distances defined by limits of main river basins (blue dots/line), habitat (yellow dots/line), and lithology (red dots/line). Each scenario considered a range (2.5–1,000,000) of 23 hypothetical resistance values offered by the barrier (limit of main river basins) or the areas not occupied by the species (non-suitable habitats/lithologies). Resistance values for different scenarios (*x*-axis) are log-transformed for illustrative purposes. (**b**) Relationship between genetic differentiation (*F*_ST_) and resistance distances calculated using Circuitscape for the best fitting scenario (resistance offered by the boundaries of main river basins set to 100; see Table 3).

Genetic differentiation was significantly and positively correlated with resistance distances obtained under all tested scenarios (Supplementary Table S3). Hypothetical scenarios based on habitat and lithology reached the highest model fit at the lowest resistance value for the non-suitable category, indicating that they do not explain the data better than a flat landscape in which all cells have equal resistance (=1) (Supplementary Table S3; Fig. 5a). In contrast, model fit for the scenario incorporating the resistance offered by the boundaries of main river basins peaked when the resistance value offered by this landscape feature was set to 100 (*r*^2^ = 0.830; *P* = 0.001) (Supplementary Table S3; Fig. 5a). A multiple matrix regression with randomization (MMRR) analysis considering simultaneously the best fit resistance value under each scenario showed that the scenario incorporating the resistance offered by the boundaries of main river basins was the only one retained into the final model (Table 3; Fig. 5b). This indicates that isolation in different river basins is the main factor explaining genetic differentiation in the species, with no apparent effect of topographic roughness, lithology or habitat (Table 3).

**Table 3:** Multiple matrix regression with randomization (MMRR) for genetic differentiation (*F*_ST_) in relation with resistance distances defined by (i) a flat landscape, (ii) topographic roughness (slope), (iii) limits of main river basins, (iv) habitat, and (v) lithology.

Variable	β	*t*	*p*
Explanatory terms			
Constant	−0.246	−2.117	0.067
Limits of main river basins (100)	1.382	7.973	0.001
Rejected terms			
Lithology (2.5)		0.648	0.827
Habitat (2.5)		2.366	0.312
Topography (slope)		0.304	0.836
Flat landscape		−1.102	0.257

The last three scenarios initially considered a range (2.5–1,000,000) of hypothetical resistance values offered by the barrier (limit of main river basins) or the areas not occupied by the species (non-suitable habitats/lithologies), but only resistance values (indicated in parentheses) yielding their respective best fitting models were included in this multivariate analysis (see Supplementary Table S3).

## Discussion

Genomic data revealed that populations of the endangered Iberian grasshopper *D*. *crassiusculus* show a marked hierarchical genetic structure, with the presence of two highly divergent cryptic lineages (Fig. 3) that comprise three genetic clusters (Figs 1 and 2). One of the lineages is only represented by the highly isolated population (ORCE) located in the southernmost limit of species distribution (Southern cluster), whereas the other includes the remainder of the populations and is sub-structured into two genetic clusters (Northern and Central clusters). Our phylogenomic and coalescent-based analyses supported an early split of the two lineages and estimated that their divergence took place during the Upper Pleistocene (~126 ka), probably around the Eemian interglacial stage. The Northern (TAJU-BELI) and Central (SALI-PHUE-BONI) genetic clusters were estimated to diverge much more recently (~17 ka), probably after the LGM. Note, however, that these estimates of divergence time must be interpreted with caution. In particular, it is remarkable the different estimates of divergence time obtained for Model D *vs*. Models A–B (Table 2). The fact that Model D does not consider gene flow among contemporary populations is expected to have resulted in younger estimates of population split than in Models A–B. Thus, Model D and Models A–B are statistically indistinguishable but find two different solutions that fit equally well our genomic data (Models A–B: presence of contemporary gene flow and older estimates of divergence times; Model D: lack of contemporary gene flow and younger estimates of divergence times) (Tables 1 and 2). Statistical evaluation of alternative migration models showed that the most likely scenarios were always those considering ancient gene flow between ancestral populations and contemporary gene flow between populations from Northern and Central genetic clusters, although with very low absolute values for migration rates per generation (*m*_ANC_ = 1.30 × 10^−05^; *m*_N-C_ = 3.47 × 10^−05^; Table 2 and Fig. 4). The consistent support for models including gene flow between ancestral populations (*m*_ANC_), indicate that vicariance with multiple contacts (probably during glacial-interglacial cycles) is likely to have led to the current genetic structure of the species (i.e. isolation with gene flow). The best supported scenario (Model B) is the one considering gene flow between recently split populations across time, with higher contemporary migration rates among closer populations from Northern and Central genetic clusters (Fig. 4). Migration models involving gene flow with the Southern lineage were either not supported (Model C) or yielded point estimates of migration rates an order of magnitude lower (*m*_C-S_ = 1.83 × 10^−06^) than those inferred between Northern and Central populations (Model A) (Table 1; Fig. 4). These results are in agreement with Bayesian clustering analyses, which showed considerable genetic admixture (~25%) among nearby populations from Northern and Central genetic clusters but no signature of admixed ancestry for the Southern lineage (Fig. 1d). Thus, despite the small distribution range and the relatively short geographical distances separating the extant populations of *D*. *crassiusculus*, our results indicate that this species shows a remarkable genetic structure that is comparable to that reported for other Orthoptera taxa with patchy distributions and forming highly fragmented populations^27,28,51,52^.

Our landscape genetic analyses indicate that geographical distance, the spatial distribution of suitable habitats, lithology or topography do not explain *per se* the degree of genetic differentiation among populations and revealed that the limits of major river basins are the main factor explaining large-scale patterns of genetic structure in *D*. *crassiusculus* (Fig. 1a). These results are in agreement with inferences from Structure and PCA analyses, which showed that the populations of the species are clustered according to the limits of main river basins: Northern genetic cluster in Tagus river basin, Central genetic cluster in Guadiana river basin, and Southern genetic cluster in Guadalquivir river basin (Fig. 1a). Apart from numerous freshwater fishes^53,54^, the importance of palaeo- and modern drainages in structuring genetic variation has been also reported in another steppe specialist grasshopper (*Mioscirtus wagneri*) presenting highly fragmented populations and inhabiting a similar geographic area^51^ and in geckos (genus *Rhynchoedura*) from arid regions of Australia^55^. These results indicate the importance of this landscape feature on the evolutionary histories of terrestrial organisms from steppe and arid landscapes^55^. Rivers themselves do not seem to be an important barrier to dispersal in our study system, as populations located within the same basin but at different sides of main river stems or their tributaries (e.g. TAJU and BELI) show low levels of genetic differentiation in comparison with populations located in different basins^56^. Estimates of divergence time among contemporary populations of *D*. *crassiusculus* (~17–126 ka; Table 2) and the timing of species split from its sister taxon *D*. *kraussi* (1.01 Ma^48^) indicate that the origin of the species and its different lineages is probably posterior to the formation of the main river basins from the central-south Iberia, which are thought to have acquired their current configuration during the Oligocene-Pliocene^54,55,57^. Thus, the different genetic clusters and lineages are not likely to have resulted from population isolation in different palaeodrainages or ancient geological surfaces^26^, but probably reflect the role of river drainages and lowlands as corridors of suitable habitat facilitating connectivity among populations located within the same basin^51^. Given that a predominantly flat landscape characterizes the distribution area of *D*. *crassiusculus* and the main drainages are not separated by an abrupt topography (i.e. mountain systems), our results suggest that populations of the species have probably remained linked to lowlands (e.g. pseudo-steppe saline low grounds) from different river basins^51,58^ rather than physically separated by ridges representing the divides between drainages. Accordingly, our analyses indicate that other landscape features such as topographic roughness (slope) or the distribution of the typical habitats and lithological formations occupied by the species are not important factors explaining spatial patterns of genetic structure in *D*. *crassiusculus* (Table 3). Previous studies have identified topographic roughness as a relevant factor shaping genetic differentiation in two montane grasshoppers inhabiting areas with abrupt landscapes^25,59^, a situation contrasting with the predominantly flat areas characterizing the distribution range of *D*. *crassiusculus*^3^. The widespread presence of sedimentary lithologies (evaporites, limestones, and conglomerates) across the distribution range of the species could have reduced our ability to identify barriers to dispersal linked to unsuitable geological formations or, alternatively, might reflect the capacity of the species to cross them. In any case, we must point out that our landscape genetic analyses should be interpreted with extreme caution, given that the very few extant populations of the species (*n* = 6) strongly limit the power of our analyses and the scope of the obtained inferences.

Coalescent-based analyses support the fact that range-wide patterns of genetic structure in *D*. *crassiusculus* are a consequence of ancient processes of population fragmentation (~17–126 ka; Table 2) that predate the Anthropocene. Accordingly, landscape genetic analyses suggest that land clearing for agriculture is not likely to explain large-scale patterns of genetic fragmentation (Fig. 5; Table 3). Based on the degree of divergence between the different lineages and genetic clusters, we recommend that the Northern, Central and Southern groups are recognized as Evolutionarily Significant Units (ESU)^60^, Designatable Units (DU)^61^ or Conservation Significant Units (CSU)^62^. These entities are likely to be substantially reproductively isolated from each other, represent an important component in the evolutionary legacy of the species, and include all discrete genetic and geographic subunits below the species level for status assessment, establishing conservation priorities and setting on-ground management strategies^60,63^. Of particular concern is the highly divergent Southern lineage because, as far as we know, it is currently represented by a single small population (ORCE) within the Guadalquivir river basin and Andalucía region (Fig. 1a,d). The correspondence between the identified units (lineages and genetic clusters) and the circumscription of different government administrations (Madrid, Castilla-La Mancha and Andalucía regions) could facilitate the establishment of regional conservation plans aimed at implementing the most efficient management strategies within each territory. Although nearby populations (TAJU-BELI and PHUE-SALI) showed no apparent signatures of genetic fragmentation (Fig. 1d), we must point that several lines of evidence suggest that this finding is not incompatible with a dramatic impact of human activities on the decline of the species at local and regional scales^3^. For instance, historical museum records indicate that many populations from the Northern cluster (specifically, in Madrid province) have been extirpated in the last decades^3,49^. All remaining populations are extremely small and submitted to severe impacts of human intervention (e.g. land ploughing, urbanization) and stochastic phenomena (e.g. flash flooding) that have been linked to sharp population declines^3,49^. The expected time-lag between population fragmentation/declines and the genetic consequences of such processes (disruption of gene flow, genetic differentiation, loss of genetic diversity, etc.) might explain why recent human impacts have not been yet reflected in spatial patterns of genetic variation^64^. Unfortunately, the small number of extant populations at local/regional scales (1–2 populations/genetic cluster), makes difficult to perform detailed analyses to evaluate the role of current landscape structure (e.g. land clearing for agriculture, urbanization, etc.) on the genetic connectivity of contemporary populations^27,65^. Future genomic analyses of specimens available in museum collections^3^ could help to determine temporal changes in genetic diversity and study past patterns of gene flow in relation with historical landscape composition^52^.

Overall, our genomic data support that the different lineages and genetic clusters of *D*. *crassiusculus* can be regarded as independent units that require adequate conservation and management strategies to preserve their idiosyncratic evolutionary histories. Conservation actions for *D*. *crassiusculus* should be focused on the preservation of areas with sensitive habitat occupied by the main lineages and units delineated by our genomic analyses. These should include the control of negative human interventions and the monitoring of local populations, actions that could also benefit other co-distributed and poorly-known species with similar ecological requirements and fragmented populations linked to gypsum and salt steppes of the Iberian Peninsula^7,10,27,66,67^. Given the extremely low number and size of extant populations of the species, *ex-situ* conservation plans and reintroduction/translocations programmes in restored habitats could help to reduce the chances of species/lineage extinction^68,69^. These conservation actions should always consider the genomic singularity of the different units identified in this study and be accompanied with long-term habitat management and population monitoring^68,70^. Future studies including detailed ecological information (e.g. diet analyses^71^) and genome scans to detect potential loci under selection implicated in ecological adaptation^72,73^ would be of great help to get a better understanding of the processes underlying the evolutionary history of the different lineages and refine the conservation actions for this endangered species.

## Methods

### Study area and sampling

During 2008–2015, we sampled six populations of *Dociostaurus crassiusculus* (Pantel, 1886) (Fig. 1; Table 4). All the populations were found in areas with a particular lithological composition (evaporites, limestones, and conglomerates) and with plant communities linked to gypsum or hypersaline soils. We are confident that these populations cover the entire distribution range of the species, as other areas with potentially adequate habitats (i.e. pseudo-steppe saline grounds, wastelands with halophytic vegetation and surroundings of hypersaline/saline lagoons with marl-gypsum outcrops) have been extensively prospected without any records of the species^3,48^. *Dociostaurus crassiusculus* has been recently assigned to the category “endangered” in the IUCN red list of threatened species due to the high fragmentation of its very small size populations^3,44^ and, for this reason, we only collected 5–6 adult individuals per population. We aimed at collecting an equal number of males and females in each locality, but samples sizes are often male-biased due to very low female numbers in some populations. Monitoring of some of the studied populations indicates that the abundance of *D*. *crassiusculus* in years before and after sampling was qualitatively similar, which suggests that the removal of only 5–6 individuals per locality had little impact on the population dynamics of the species. Fresh whole specimens were stored in 2,000 µL ethanol 96% at −20 °C until used for genomic analyses.

**Table 4:** Geographical location and number of samples (*n*) for the studied populations of the Iberian cross-backed grasshopper *Dociostaurus crassiusculus*.

Locality	Province	Code	*n* males	*n* females	Latitude	Longitude
Perales de Tajuña	Madrid	TAJU	4	1	40.2064	−3.3181
Belinchón	Cuenca	BELI	3	3	40.0793	−3.0446
Laguna de Peña Hueca	Toledo	PHUE	3	3	39.5158	−3.3486
Laguna de Salicor	Ciudad Real	SALI	3	3	39.4637	−3.1808
El Bonillo	Albacete	BONI	3	3	38.8779	−2.4834
Orce	Granada	ORCE	3	3	37.7515	−2.4259

### DNA extraction and genomic library preparation

We used NucleoSpin Tissue kits (Macherey-Nagel, Durën, Germany) to extract and purify genomic DNA from the hind femur of each individual. Genomic DNA was individually barcoded and processed into one genomic library using the double-digestion restriction-fragment-based procedure (ddRADSeq) described in Peterson, *et al*.^74^. In brief, DNA was doubly digested with the restriction enzymes *Mse*I and *Eco*RI (New England Biolabs, Ipswich, MA, USA) and Illumina adaptors including unique 7-bp barcodes were ligated to the digested fragments. Ligation products were pooled, size-selected between 475–580 bp with a Pippin Prep (Sage Science, Beverly, MA, USA) machine and amplified by PCR with 12 cycles using the iProof^TM^ High-Fidelity DNA Polymerase (BIO-RAD, Hercules, CA, USA). The library was sequenced in a single-read 150-bp lane on an Illumina HiSeq. 2500 platform at The Centre for Applied Genomics (SickKids, Toronto, ON, Canada).

### Genomic data processing and bioinformatics

We used both Stacks v. 1.35^75–77^ and PyRAD v. 3.0.66^78^ to assemble our sequences into *de novo* loci and call genotypes. This allowed us to examine the robustness of our analyses based on SNP datasets obtained using two of the most popular programs currently available to assemble RAD-seq data^76,78^. The choice of different filtering thresholds using either Stacks or PyRAD had little impact on the obtained inferences^50^. For this reason, unless otherwise indicated, all downstream analyses were performed using a SNP dataset obtained with Stacks including only those loci that were represented in at least four populations (*p* = 4). See Supplementary Methods for additional details on sequence assembling and data filtering.

### Population genetic statistics

Population genetics statistics, including major allele frequency (*P*), nucleotide diversity (*π*), observed (*H*_O_) and expected (*H*_E_) heterozygosity, and the Wright’s inbreeding coefficient (*F*_IS_), were calculated using the program *populations* from Stacks^75^. For biallelic RADSeq loci, *π* is an estimate of expected heterozygosity and is therefore a useful measure of the genetic diversity of populations. Furthermore, *F*_IS_ measures the reduction in observed heterozygosity as compared to expected heterozygosity for an allele in a population, with positive values indicating non-random mating or cryptic population structure^79–82^. Pair-wise *F*_ST_ values of genetic differentiation were calculated between all pairs of populations in Arlequin v.3.5^83^. We used PGDSpider v. 2.1.0.3^84^ to convert Variant Call Format (VCF) files provided by Stacks into the correct format needed for Arlequin.

### Population genetic structure

We analysed population genetic structure and identified groups of individuals with similar ancestral gene pools using the Bayesian clustering method implemented in the program Structure v.2.3.3^85–87^. We ran Structure using a random subset of 10,000 unlinked SNPs from six different datasets obtained with Stacks and PyRAD considering different filtering/clustering parameters (see Supplementary Methods for further details). For each dataset, we ran Structure assuming correlated allele frequencies and admixture and without using prior population information^86^. We conducted 15 independent runs for each value of *K*, where *K* ranged from 1 to *n* + 1 for the dataset of *n* populations, to estimate the “true” number of clusters with a burn-in step of 100,000 iterations followed by 200,000 MCMC cycles. We retained the ten runs having the highest likelihood for each value of *K* and defined the number of populations best fitting the dataset using log probabilities [Pr(X|*K*)]^87^ and the Δ*K* method^88^, as implemented in Structure Harvester^89^. We used Clumpp v. 1.1.2 and the Greedy algorithm to align multiple runs of Structure for the same *K* value^90^ and Distruct v. 1.1^91^ to visualize as bar plots the individual’s probabilities of membership to each inferred genetic cluster. Complementary to Bayesian clustering analyses and in order to visualize the major axes of population genetic differentiation, we performed individual-based PCA using the R 3.3.3 (R Core Team 2017) package Adegenet^92^.

### Phylogenomic inference

We inferred the phylogenetic relationships among the studied populations using the coalescent model implemented in the Snapp v.1.3.0^93^ plug-in for Beast v.2.4.5^94^. Due to the large computational demands of this program, Snapp analyses were conducted using a random subset of 2,500 SNPs and including four populations (TAJU, PHUE, BONI, and ORCE) representative of the main geographical areas (i.e. populations separated >80 km; Fig. 1a) and the three genetic groups identified by PCA and Bayesian clustering analyses in Structure (Northern, Central and Southern clusters) (see Results section). We ran these analyses using different theta priors to allow for different current and ancestral population sizes (scenario 1: *α* = 2; *β* = 200; and scenario 2: *α* = 2; *β* = 20,000). The forward (*u*) and reverse (*v*) mutation rates were set to be calculated by Snapp and the remaining parameters were left at default values. We used the phrynomics R script written by Barb Banbury (https://github.com/bbanbury/phrynomics) to remove non-binary and invariant SNPs, code heterozygotes, and format input files for Snapp. We used different starting seed numbers to run two independent runs for each scenario, each with >5 million generations sampled every 1,000 steps. Each run was inspected in Tracer v.1.6^95^ in order to check the convergence to stationary of the chains and confirm that Effective Sample Sizes (ESS) for all parameters were always much higher than 200. Afterwards, we combined the two replicate runs for each analysis using LogCombiner v.2.4.5, discarded 10% of trees as burn-in and used TreeAnnotator v.2.4.5 to obtain maximum credibility trees. Phylogenetic trees were displayed with DensiTree v.2.2.5^96^. Complementary to Snapp, we also ran phylogenetic analyses using SVDquartets^97^ as implemented in Paup* v.4.0a152^98^. Analyses with SVDquartets included *Dociostaurus maroccanus* (Thunberg, 1815) as outgroup. Phylogenetic trees were constructed by exhaustively evaluating all possible quartets from the dataset and uncertainty in relationships was quantified using 1,000 bootstrapping replicates.

### Coalescent-based demographic models

We used Fastsimcoal2 and the site frequency spectrum (SFS)^99,100^ to compare six hypothetical models of gene flow (see Fig. 4), calculate the composite likelihood of the probability of the observed data given a specified model, and estimate divergence times (*t*), effective population sizes (θ), and migration rates per generation (*m*)^99,100^ under the best supported model/s. For Fastsimcoal2 analyses we considered the three genetic groups inferred by Structure and PCAs (Northern, Central and Southern) and the topology yielded by phylogenomic analyses in Snapp (see Results section)^81,101^. For each of the three population groups considered in the simulations, we selected 11 individuals from the Northern cluster, 12 individuals from the Central cluster, and the 6 individuals from the Southern cluster. A folded joint SFS was calculated considering a single SNP per locus to avoid the effects of linkage disequilibrium^102^. Because we did not consider invariable sites in the SFS (i.e. “removeZeroSFS” option in Fastsimcoal2), we fixed the effective population size for one of the populations (ORCE; θ_S_) to enable the estimation of other parameters in Fastsimcoal2^50,81,99,102^. The effective population size fixed in the models was calculated from the level of nucleotide diversity (*π*) and estimates of mutation rate per site per generation (*μ*), since Ne = (*π*/4*μ*). Nucleotide diversity (*π*) for the population ORCE was estimated from polymorphic and non-polymorphic loci using Stacks (*π* = 0.0011; Supplementary Table S1). We considered an average mutation rate per site per generation^102,103^ of 3.50 × 10^−9^. To remove all missing data for the calculation of the joint SFS and minimize errors with allele frequency estimates, each population group was downsampled to 8–4 individuals (Northern group: 7 individuals; Central group: 8 individuals; Southern group: 4 individuals) using a custom Python script written by Qixin He and available on Dryad^102^. The final SFS contained information for 10,167 variable SNPs.

Each of the six models was run 100 replicated times using the computing resources provided by CESGA (Galician Supercomputer Center, Spain) and considering 100,000–250,000 simulations for the calculation of the composite likelihood, 10–40 expectation-conditional maximization (ECM) cycles, and a stopping criterion of 0.001^50,102^. We used an information-theoretic model selection approach based on the Akaike’s information criterion (AIC) to determine the probability of each model given the observed data^104–106^. After the maximum likelihood was estimated for each model, we calculated the AIC scores^106^. AIC values for each model were rescaled (ΔAIC) calculating the difference between the AIC value of each model and the minimum AIC obtained among all competing models (i.e. the best model has ΔAIC = 0). Confidence intervals of parameter estimates for the best supported models were obtained from 100 parametric bootstrap replicates by simulating SFS from the maximum composite likelihood point estimates and re-estimating parameters each time^81^.

### Landscape genetic analyses

We generated alternative spatially-explicit isolation-by-resistance (IBR) scenarios of population connectivity and tested which one is better supported by observed data of genetic differentiation^107^. We applied circuit theory and used Circuitscape 4.0^108,109^ to calculate resistance distance matrices between all pairs of populations under five hypothetical scenarios of gene flow: (i) a “flat” landscape in which all cells have equal resistance (resistance = 1), which is analogous to geographical distance but more appropriate for comparison with others competing models also generated with Circuitscape^25^; (ii) topographic roughness (slope); (iii) resistance offered by the boundaries of the main river basins from the study area (Tagus, Guadiana, Guadalquivir, Júcar, and Segura rivers; Fig. 1) (iv) resistance offered by non-natural landscapes and natural habitats not occupied by the species; and (v) resistance offered by areas with lithologies where the species is not present. Topographic roughness (slope) was calculated using a 90-m resolution digital elevation model from NASA Shuttle Radar Topographic Mission (SRTM Digital Elevation Data; http://srtm.csi.cgiar.org/) and the final layer was transformed to 30 arc-sec (c. 1 km) resolution for subsequent analyses. Natural habitats occupied by the species, natural habitats not occupied by the species, and non-natural habitats were defined according to Corine Land Cover maps^110^. We considered as natural habitats occupied by the species the Corine Land Cover categories “Natural grassland” and “Sclerophyllous vegetation”, which represent the two habitat classes used by the species according to our own occurrence data^3^. Natural habitats not occupied by the species included all other habitats falling within the category “Forest and semi-natural areas” plus the category “Pastures”. Non-natural habitats not occupied by the species grouped all other land cover categories, including agricultural areas and artificial surfaces^110^. The lithological categories constituting the typical habitats occupied by the species (evaporites, limestones, and conglomerates) were identified according to our own occurrence data^3^ and mapped using the spatial dataset OneGeology-Europe (http://info.igme.es/cartografia/oneGeology.asp?mapa = oneGeology). In scenarios iii-v we assigned a range of resistance values (2.5–1,000,000) to the barrier (limit of main river basins) or the areas not occupied by the species (non-suitable habitats/lithologies), which allowed us to identify the resistance value for these landscape features that best fits our data of genetic differentiation (*F*_ST_)^27,111^. Non-natural habitats (agricultural areas and artificial surfaces) were assumed to offer twice the resistance than natural habitats not occupied by the species (Supplementary Table S3). Background areas (i.e. areas within main river basins and habitats/lithologies occupied by the species) were given a fixed value of 1. All maps and GIS calculations were performed using ArcMap v.10.2.1 (ESRI, Redlands, CA, USA). In Circuitscape, we employed a four-neighbor cell connection scheme in order to make effective the resistance assigned to river basin boundaries, as linear landscape features become permeable through pixel corners under the eight-neighbor cell connection scheme^108^. Finally, we determined how well the different landscape resistance models fit observed data of genetic differentiation (*F*_ST_) using multiple matrix regressions with randomization (MMRR) as implemented in R 3.3.3^107^. The final model was selected following a backward procedure, initially fitting all explanatory terms and progressively eliminating non-significant variables until all retained variables were significant. The significance of the variables excluded from the model was tested again until no additional variable reached significance^67^.

## Electronic supplementary material

Supplementary Information

## Data Availability

SNP datasets and all other data generated and analysed in this study are available in Figshare or included in the published article and its Supplementary Information file.
