# Supplementary material for: Using high-throughput sequencing to investigate the factors structuring genomic variation of a Mediterranean grasshopper of great conservation concern
Source: Sci Rep. 2018 Sep 7;8:13436. doi: 10.1038/s41598-018-31775-x (PMC6128945; doi:10.1038/s41598-018-31775-x)
Supplement: Supplementary file 1 — Supplementary Information [file 41598_2018_31775_MOESM1_ESM.docx]

**Using high-throughput sequencing to investigate the factors structuring genomic variation of a Mediterranean grasshopper of great conservation concern**

María José González-Serna*^1^, Pedro J. Cordero^1^, and Joaquín Ortego^2^

^1^ Grupo de Investigación de la Biodiversidad Genética y Cultural, Instituto de Investigación en Recursos Cinegéticos – IREC – (CSIC, UCLM, JCCM), Ronda de Toledo, 12, E-13071 Ciudad Real, Spain.

^2^ Department of Integrative Ecology, Estación Biológica de Doñana – EBD – (CSIC), Avda. Américo Vespucio, 26, E-41092 Seville, Spain.

*** Author for correspondence:**

María José González-Serna

Grupo de Investigación de la Biodiversidad Genética y Cultural

Instituto de Investigación en Recursos Cinegéticos – IREC – (CSIC, UCLM, JCCM)

Ronda de Toledo, 12, E-13071 Ciudad Real, Spain.

E-mail: mariajose.gonzalez@uclm.es

Tel: +34 926 29 54 50

Fax: +34 926 59 54 51


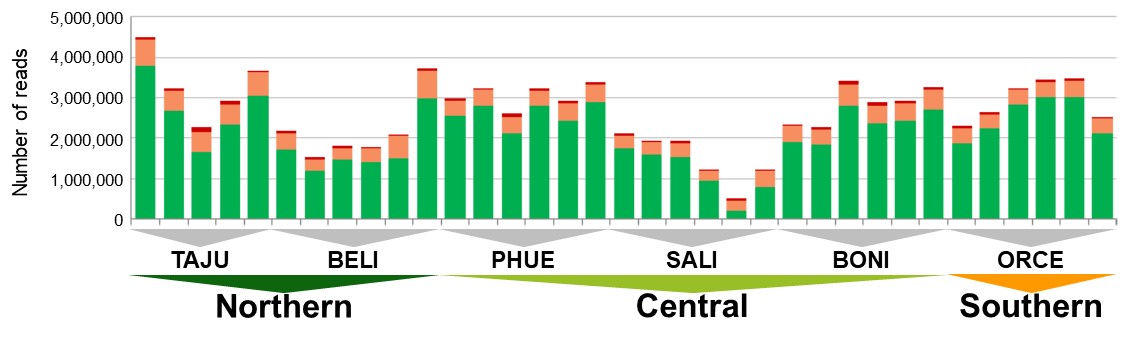
**Supplementary Figure S1** Number of reads per individual before and after different quality filtering steps by Stacks. The total height of the bars represents the total number of raw reads obtained for each individual. Within each bar, the dark red color represents the reads that were discarded by *process_radtags* due to low quality, adapter contamination or ambiguous barcode and orange color represents the reads that were discarded by *ustacks* after filtering out repetitive elements and reads that did not comply the different criteria required to create a “stack”. Green color represents the number of retained reads used to identify homologous loci. Populations are sorted from NW to SE and are labelled using the same codes presented in Table 4.

**Supplementary Figure S2** Results of Bayesian clustering analyses in Structure based on a random subset of 10,000 SNPs obtained with Stacks for *p* = 2 (a, c) and *p* = 4 (b, d). Panels a) and b) show the mean (± SD) log probability of the data (LnPr (X|*K*) over 10 best runs (left *y*-axis, black dots and error bars) for each value of *K*. The magnitude of Δ*K* as a function of *K* (right *y*-axis, open dots) indicates the best-supported number of clusters (*K* = 2 in all cases). Panels c) and d) show the individual’s probabilities of membership to each inferred genetic cluster from *K* = 2 to *K* = *n* (*n*: number of populations). Each individual is represented by a vertical bar, which is partitioned into *k* coloured segments showing the individual’s probability of belonging to the cluster with that colour. Thin vertical black lines separate individuals from different populations.


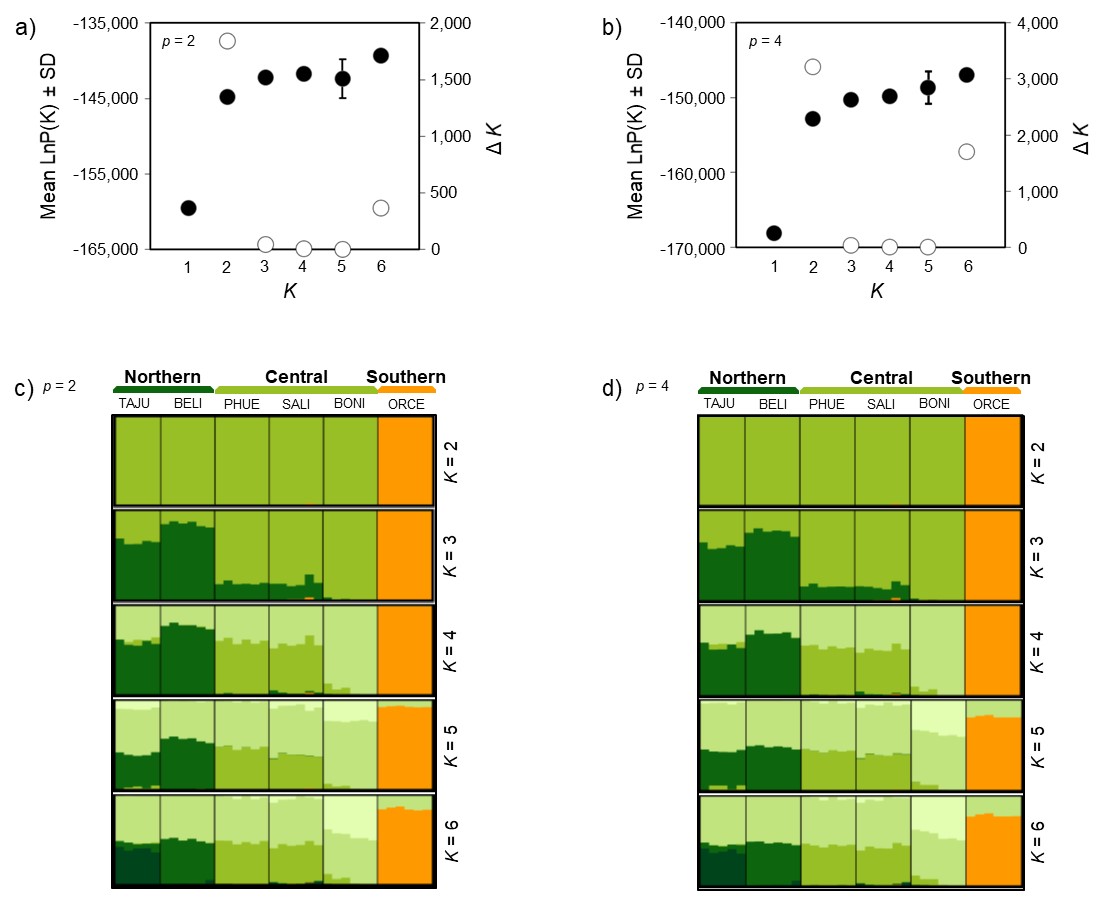


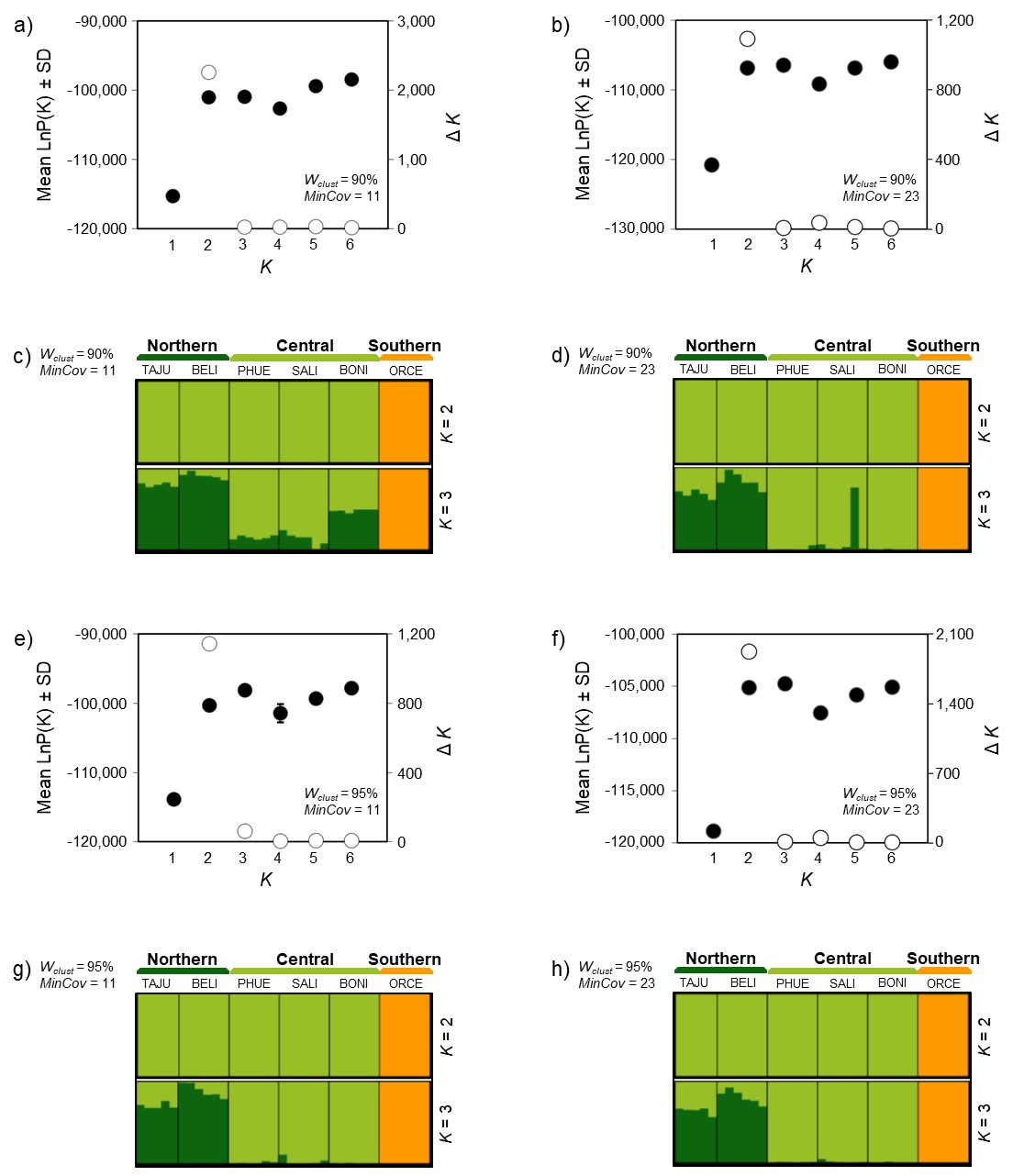
**Supplementary Figure S3** Results of Bayesian clustering analyses in Structure based on a random subset of 10,000 SNPs obtained with PyRad using two different clustering thresholds of sequence similarity (*W_clust_* = 90% and 95%) and two different values of minimum taxon coverage in a given locus (*MinCov* = 11 and 23). Panels a), b), e) and f) show the mean (± SD) log probability of the data (LnPr (X|*K*) over 10 best runs (left *y*-axis, black dots and error bars) for each value of *K*. The magnitude of Δ*K* as a function of *K* (right *y*-axis, open dots) indicates the best-supported number of clusters (*K* = 2 in all cases). Panels c), d), g) and h) show the individual’s probabilities of membership to each inferred genetic cluster for *K* = 2 and *K* = 3. Each individual is represented by a vertical bar, which is partitioned into *k* coloured segments showing the individual’s probability of belonging to the cluster with that colour. Thin vertical black lines separate individuals from different populations.

**Supplementary Figure S4** Principal component analyses (PCA) of genetic variation for populations of *D. crassiusculus*. Panels a) and b) show analyses based on SNP datasets obtained with Stacks considering two different filtering parameters (*p* = 2 and *p* = 4). Panels c), d), e) and f) show analyses based on SNP datasets obtained with PyRad using two different clustering thresholds of sequence similarity (*W_clust_* = 90% and 95%) and two different values of minimum taxon coverage in a given locus (*MinCov* = 11 and 23). Dotted-line rectangles group main population clusters. Population codes are described in Table 4.

**
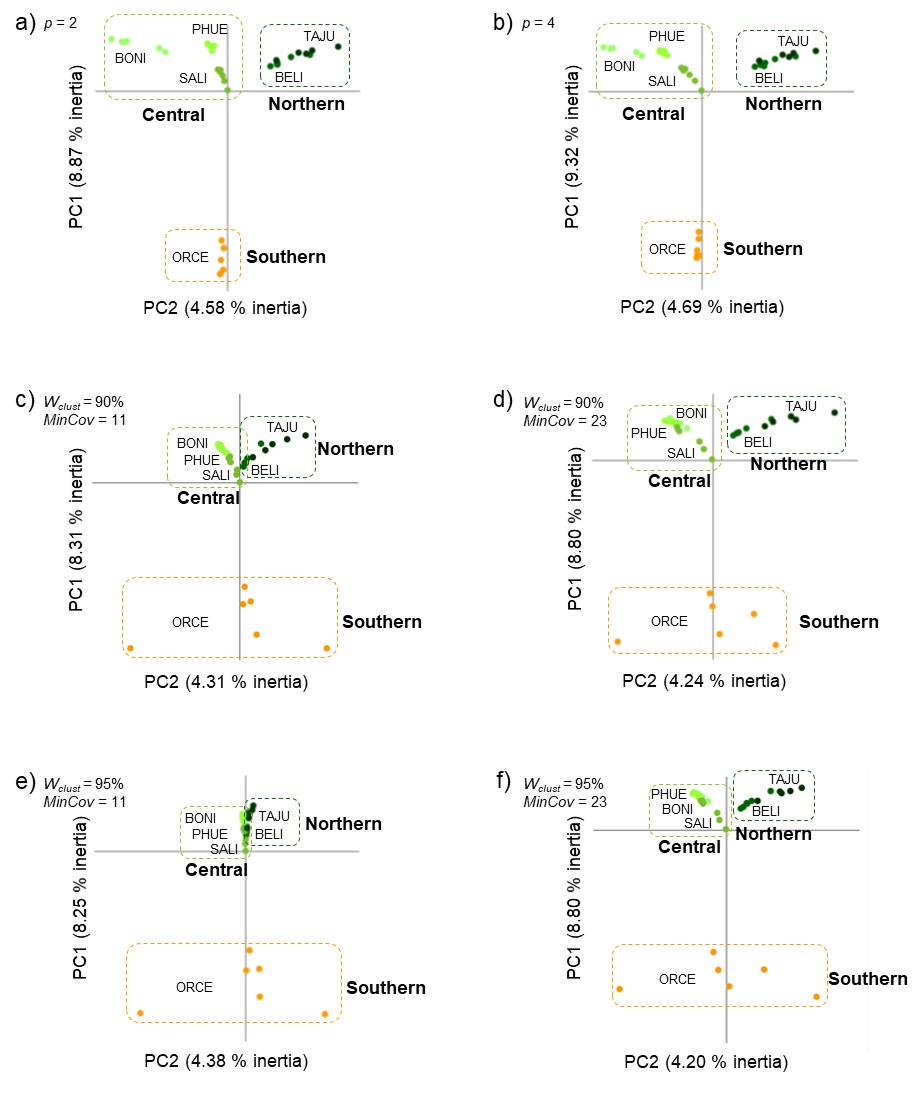
**

**Supplementary Table S1** Population genetic statistics (*P*, π, *H*_O_, *H*_E_, and *F*_IS_) for the studied populations of the Iberian cross-backed grasshopper *Dociostaurus crassiusculus*. Average values across loci are presented for major allele frequency (*P*), nucleotide diversity (*π*), observed (*H*_O_) and expected (*H*_E_) heterozygosity, and the Wright’s inbreeding coefficient (*F*_IS_). Genetic statistics were calculated in Stacks for all positions (polymorphic and non-polymorphic) and considering loci that were represented in at least two (*p* = 2) or four populations (*p* = 4) and the 50% of individuals within populations (*r* = 0.5).

| Code | *P* | | π | | *H*_O_ | | *H*_E_ | | *F*_IS_ | |
| --- | --- | --- | --- | --- | --- | --- | --- | --- | --- | --- |
|  | *p* = 2 | *p* = 4 | *p* = 2 | *p* = 4 | *p* = 2 | *p* = 4 | *p* = 2 | *p* = 4 | *p* = 2 | *p* = 4 |
| TAJU | 0.9992 | 0.9992 | 0.0013 | 0.0012 | 0.0009 | 0.0009 | 0.0011 | 0.0011 | 0.0007 | 0.0006 |
| BELI | 0.9992 | 0.9993 | 0.0012 | 0.0011 | 0.0009 | 0.0009 | 0.0010 | 0.0010 | 0.0005 | 0.0004 |
| PHUE | 0.9992 | 0.9992 | 0.0013 | 0.0012 | 0.0009 | 0.0009 | 0.0011 | 0.0011 | 0.0007 | 0.0006 |
| SALI | 0.9993 | 0.9993 | 0.0012 | 0.0011 | 0.0009 | 0.0009 | 0.0010 | 0.0010 | 0.0004 | 0.0004 |
| BONI | 0.9992 | 0.9993 | 0.0012 | 0.0011 | 0.0009 | 0.0009 | 0.0010 | 0.0010 | 0.0006 | 0.0005 |
| ORCE | 0.9992 | 0.9993 | 0.0012 | 0.0011 | 0.0009 | 0.0009 | 0.0010 | 0.0010 | 0.0006 | 0.0005 |

**Supplementary Table S2** Pairwise *F*_ST_ values calculated in Arlequin. All *F*_ST_ values are significantly different from zero after sequential Bonferroni corrections (*P* < 0.05).

| Code | TAJU | BELI | PHUE | SALI | BONI | ORCE |
| --- | --- | --- | --- | --- | --- | --- |
| TAJU | -- |  |  |  |  |  |
| BELI | 0.063 | -- |  |  |  |  |
| PHUE | 0.071 | 0.099 | -- |  |  |  |
| SALI | 0.074 | 0.107 | 0.033 | -- |  |  |
| BONI | 0.105 | 0.125 | 0.088 | 0.093 | -- |  |
| ORCE | 0.206 | 0.233 | 0.212 | 0.192 | 0.237 | -- |

**Supplementary Table S3** Multiple Matrix Regressions with Randomization (MMRR) for genetic differentiation (*F*_ST_) in relation with resistance distances defined by (i) a flat landscape, (ii) topographic roughness (slope), (iii) limits of main river basins, (iv) habitat, and (v) lithology. The last three scenarios considered a range of hypothetical resistance values offered by the barrier (limit of main river basins) or the areas not occupied by the species (non-suitable habitats/lithologies). Non-natural habitats (agriculture and artificial surfaces) were assumed to offer twice the resistance than natural habitats not occupied by the species (asterisks) (see Methods for further details). The best-supported scenario is indicated in bold.

| Model | Resistance values | *R*^2^ | β | t | p |
| --- | --- | --- | --- | --- | --- |
| Flat landscape | - | 0.739 | 1.784 | 6.074 | 0.005 |
| Topography | - | 0.657 | 0.998 | 4.987 | 0.033 |
| Limits of main river basins | 2.5 | 0.751 | 1.764 | 6.259 | 0.006 |
| Limits of main river basins | 5 | 0.762 | 1.740 | 6.460 | 0.006 |
| Limits of main river basins | 10 | 0.778 | 1.703 | 6.750 | 0.006 |
| Limits of main river basins | 20 | 0.798 | 1.645 | 7.156 | 0.004 |
| Limits of main river basins | 30 | 0.810 | 1.597 | 7.437 | 0.004 |
| Limits of main river basins | 40 | 0.818 | 1.555 | 7.638 | 0.003 |
| Limits of main river basins | 50 | 0.823 | 1.519 | 7.780 | 0.003 |
| Limits of main river basins | 75 | 0.830 | 1.443 | 7.956 | 0.002 |
| **Limits of main** **river basins** | **100** | **0.830** | **1.382** | **7.973** | **0.001** |
| Limits of main river basins | 125 | 0.828 | 1.332 | 7.904 | 0.003 |
| Limits of main river basins | 150 | 0.824 | 1.289 | 7.789 | 0.003 |
| Limits of main river basins | 250 | 0.800 | 1.165 | 7.217 | 0.003 |
| Limits of main river basins | 500 | 0.745 | 1.001 | 6.155 | 0.002 |
| Limits of main river basins | 1,000 | 0.678 | 0.868 | 5.228 | 0.002 |
| Limits of main river basins | 2,500 | 0.606 | 0.756 | 4.470 | 0.004 |
| Limits of main river basins | 5,000 | 0.571 | 0.711 | 4.162 | 0.002 |
| Limits of main river basins | 10,000 | 0.548 | 0.684 | 3.973 | 0.004 |
| Limits of main river basins | 25,000 | 0.527 | 0.663 | 3.807 | 0.002 |
| Limits of main river basins | 50,000 | 0.515 | 0.652 | 3.712 | 0.025 |
| Limits of main river basins | 100,000 | 0.505 | 0.644 | 3.642 | 0.021 |
| Limits of main river basins | 250,000 | 0.497 | 0.638 | 3.584 | 0.018 |
| Limits of main river basins | 500,000 | 0.494 | 0.635 | 3.561 | 0.023 |
| Limits of main river basins | 1,000,000 | 0.492 | 0.634 | 3.549 | 0.020 |
| Habitat | 2.5/1.25* | 0.682 | 1.737 | 5.283 | 0.003 |
| Habitat | 5/2.5* | 0.611 | 1.531 | 4.516 | 0.010 |
| Habitat | 10/5* | 0.545 | 1.381 | 3.948 | 0.019 |
| Habitat | 20/10* | 0.494 | 1.280 | 3.564 | 0.029 |
| Habitat | 30/15* | 0.471 | 1.237 | 3.399 | 0.040 |
| Habitat | 40/20* | 0.456 | 1.212 | 3.304 | 0.044 |
| Habitat | 50/25* | 0.447 | 1.196 | 3.240 | 0.040 |
| Habitat | 75/37.5* | 0.432 | 1.172 | 3.144 | 0.046 |
| Habitat | 100/50* | 0.423 | 1.158 | 3.090 | 0.056 |
| Habitat | 125/62.5* | 0.418 | 1.150 | 3.054 | 0.063 |
| Habitat | 150/75* | 0.414 | 1.144 | 3.029 | 0.045 |
| Habitat | 250/125* | 0.405 | 1.130 | 2.973 | 0.064 |
| Habitat | 500/250* | 0.397 | 1.119 | 2.925 | 0.061 |
| Habitat | 1,000/500* | 0.392 | 1.113 | 2.898 | 0.075 |
| Habitat | 2,500/1,250* | 0.390 | 1.109 | 2.880 | 0.077 |
| Habitat | 5,000/2,500* | 0.389 | 1.108 | 2.874 | 0.067 |
| Habitat | 10,000/5,000* | 0.388 | 1.107 | 2.871 | 0.077 |
| Habitat | 25,000/12,500* | 0.388 | 1.107 | 2.869 | 0.063 |
| Habitat | 50,000/25,000* | 0.388 | 1.107 | 2.869 | 0.068 |
| Habitat | 100,000/50,000* | 0.388 | 1.107 | 2.868 | 0.086 |
| Habitat | 250,000/125,000* | 0.388 | 1.107 | 2.868 | 0.089 |
| Habitat | 500,000/250,000* | 0.388 | 1.106 | 2.868 | 0.079 |
| Habitat | 1,000,000/500,000* | 0.388 | 1.106 | 2.868 | 0.080 |
| Lithology | 2.5 | 0.777 | 1.444 | 6.723 | 0.006 |
| Lithology | 5 | 0.763 | 1.239 | 6.469 | 0.017 |
| Lithology | 10 | 0.736 | 1.093 | 6.018 | 0.041 |
| Lithology | 20 | 0.700 | 0.990 | 5.506 | 0.081 |
| Lithology | 30 | 0.675 | 0.942 | 5.199 | 0.102 |
| Lithology | 40 | 0.657 | 0.912 | 4.987 | 0.089 |
| Lithology | 50 | 0.642 | 0.890 | 4.830 | 0.098 |
| Lithology | 75 | 0.617 | 0.855 | 4.575 | 0.126 |
| Lithology | 100 | 0.601 | 0.832 | 4.427 | 0.111 |
| Lithology | 125 | 0.591 | 0.817 | 4.335 | 0.126 |
| Lithology | 150 | 0.584 | 0.805 | 4.276 | 0.138 |
| Lithology | 250 | 0.575 | 0.777 | 4.192 | 0.115 |
| Lithology | 500 | 0.581 | 0.752 | 4.244 | 0.100 |
| Lithology | 1,000 | 0.599 | 0.735 | 4.403 | 0.070 |
| Lithology | 2,500 | 0.621 | 0.722 | 4.618 | 0.042 |
| Lithology | 5,000 | 0.633 | 0.716 | 4.739 | 0.037 |
| Lithology | 10,000 | 0.642 | 0.712 | 4.823 | 0.027 |
| Lithology | 25,000 | 0.648 | 0.710 | 4.887 | 0.025 |
| Lithology | 50,000 | 0.650 | 0.709 | 4.912 | 0.019 |
| Lithology | 100,000 | 0.651 | 0.709 | 4.925 | 0.033 |
| Lithology | 250,000 | 0.652 | 0.708 | 4.933 | 0.018 |
| Lithology | 500,000 | 0.652 | 0.708 | 4.936 | 0.028 |
| Lithology | 1,000,000 | 0.652 | 0.708 | 4.937 | 0.026 |

**Supplementary Methods**

**Genomic data processing and bioinformatics**

We used both Stacks v. 1.35 ^1-3^ and PyRAD v. 3.0.66 ^4^ to assemble our sequences into *de novo* loci and call genotypes. This allowed us to examine the robustness of our analyses based on SNP datasets obtained using two of the most popular programs currently available to assemble RAD-seq data ^2,4^. Reads were de-multiplexed and filtered for overall quality using the program *process_radtags*, retaining reads with a Phred score > 10 (using a sliding window of 15%), no adaptor contamination, and that had an unambiguous barcode and restriction cut site. Raw reads were screened for quality with Fastqc v. 0.11.5 ^5^ and all sequences were trimmed to 129-bp using Seqtk ^6^ in order to remove low-quality reads near the 3´ ends.

First, we used the different programs distributed as part of the Stacks v. 1.35 pipeline (*ustacks*, *cstacks*, *sstacks*, and *populations*) to assemble our sequences into *de novo* loci and call genotypes ^1-3^. Filtered reads of each individual were assembled *de novo* into putative loci with the *ustacks* program. The minimum stack depth (*m*) was set to three and we allowed a maximum distance of two nucleotide mismatches (*M*) to group reads into a “stack”. We used the “removal” (*r*) and “deleveraging” (*d*) algorithms to eliminate highly repetitive stacks and resolve over-merged loci, respectively. Single nucleotide polymorphisms (SNPs) were identified at each locus and genotypes were called using a multinomial-based likelihood model that accounts for sequencing errors, with the upper bound of the error rate (*ε*) set to 0.2 ^1-3^. A catalogue of loci was built using the *cstacks* program, with loci recognized as homologous across individuals if the number of nucleotide mismatches between consensus sequences (*n*) was ≤2. Each individual was matched against this catalogue using *sstacks* program and output files were exported in different formats for subsequent analyses using the program *populations*. We exported only the first SNP per RAD locus and retained loci that were sequenced in at least half of the individuals of each population (parameter *r* = 0.5) and represented in at least two (~33%; parameter *p* = 2) or four (~66%; parameter *p* = 4) populations (out of the six populations analysed; Table 4).

Second, we assembled our sequences into *de novo* loci using PyRAD v. 3.0.66 ^4^. Briefly, reads retained after *process_radtags* were further quality-filtered with PyRAD to convert base calls with a Phred score <20 into *N*s and discard reads with >2 *N*s. Retained reads were clustered within- and across samples considering two different thresholds of sequence similarity (*W*_clust_ = 90 and 95%) and clusters with a coverage depth <5 were discarded (*Mindepth* = 5). Consensus sequences with more than five heterozygous sites were excluded (*maxH* = 5) as well as loci containing one or more heterozygous sites across more than 5 samples (~15% of individuals; *maxSH* = p.15), as we expect that this represents a fixed difference among clustered paralogs rather than a true polymorphism ^4,7^. In a final filtering step, we excluded loci that were not recovered in at least 11 or 23 samples (*minCov* = 11 and 23, corresponding with ~33 and ~66% of individuals, respectively).

The choice of different filtering thresholds using either Stacks or PyRAD had little impact on the obtained inferences (see Fig. S2-4) ^7,8^. For this reason, unless otherwise indicated, all downstream analyses were performed using a SNP dataset obtained with Stacks including only those loci that were represented in at least four populations (*p* = 4).

**Supplementary references**

1. Hohenlohe, P. A. *et al*. Population genomics of parallel adaptation in *Threespine Stickleback* using sequenced RAD tags. *PLoS Genet.* **6**, e1000862 (2010).

2. Catchen, J. M., Amores, A., Hohenlohe, P., Cresko, W. & Postlethwait, J. H. Stacks: building and genotyping loci *de novo* from short-read sequences. *G3 (Bethesda)* **1**, 171-182 (2011).

3. Catchen, J., Hohenlohe, P. A., Bassham, S., Amores, A. & Cresko, W. A. Stacks: an analysis tool set for population genomics. *Mol. Ecol.* **22**, 3124-3140 (2013).

4. Eaton, D. A. PyRAD: assembly of *de novo* RADseq loci for phylogenetic analyses. *Bioinformatics* **30**, 1844-1849 (2014).

5. Simon, A. *FastQC v.0.11.7*, http://www.bioinformatics.babraham.ac.uk/projects/fastqc/ (2018).

6. Heng, L. *Seqtk*, https://github.com/lh3/seqtk (2017).

7. Eaton, D. A., Hipp, A. L., González‐Rodríguez, A. and Cavender‐Bares, J. Historical introgression among the American live oaks and the comparative nature of tests for introgression. *Evolution* **69**, 2587-2601 (2015).

8. Ortego, J., Gugger, P. F. & Sork, V. L. Genomic data reveal cryptic lineage diversification and introgression in Californian golden cup oaks (section Protobalanus). *New Phytol.* **218**, 804-818 (2018).
